# The ACCEPTance of automation: refining circulating tumor cells enumeration for improved metastatic colorectal cancer prognosis

**DOI:** 10.1002/1878-0261.70126

**Published:** 2025-09-19

**Authors:** Michela De Meo, Marco Siringo, Alessandro Vici, Ann Zeuner, Orietta Gandini, Paola Gazzaniga, Chiara Nicolazzo

**Affiliations:** ^1^ Department of Molecular Medicine Sapienza University of Rome Italy; ^2^ Medical Oncology Sant'Andrea University Hospital Rome Italy; ^3^ Department of Oncology and Molecular Medicine Istituto Superiore di Sanità Rome Italy; ^4^ Cancer Liquid Biopsy Unit, Department of Experimental Medicine Sapienza University of Rome Italy; ^5^ Department of Life Science, Health, and Health Professions Link Campus University Rome Italy

**Keywords:** ACCEPT, CellSearch^®^, circulating hybrid cells (CHC), circulating tumor cells (CTC), dual‐positive cells, metastatic colorectal cancer (mCRC)

## Abstract

Colorectal cancer (CRC) remains a leading cause of cancer‐related mortality, with metastatic CRC (mCRC) posing significant challenges due to tumor heterogeneity and resistance to therapy. Circulating tumor cells (CTC) and circulating hybrid cells (CHC) detected via liquid biopsies have emerged as promising biomarkers for monitoring disease progression. This study aimed to evaluate the prognostic utility of automated CTC enumeration using the ACCEPT software compared to a manual method and assess the potential clinical relevance of CHC in mCRC. A retrospective analysis of CellSearch^®^ images from 67 mCRC patients was conducted, correlating CTC and CHC counts with progression‐free survival and overall survival (OS). Automated enumeration demonstrated improved accuracy and reduced variability, confirming the prognostic significance of CTC counts for OS. However, CHC enumeration showed no significant association with clinical outcomes, suggesting sporadic detection rather than consistent prognostic value. These findings underscore the reliability of automated CTC enumeration in mCRC prognosis while highlighting the need for further research into the biological and clinical roles of CHC.

## Graphical abstract

This study highlights the potential of automated enumeration using the ACCEPT software to refine circulating tumor cell (CTC) detection in metastatic colorectal cancer (mCRC). The automated method demonstrates improved accuracy and reduced variability compared to the manual approach. Findings emphasize the need for improved reliability and standardization in CTC detection methods for mCRC management.

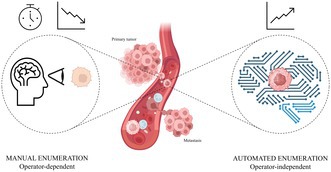

**Abbreviations**
ACCEPT: Automated CTC Classification Enumeration and PhenoTyping
APC: Allophycocyanin
AU: Arbitrary unit
CHC: Circulating hybrid cell
CI: Confidence interval
CK: Cytokeratin
CRC: Colorectal cancer
CTC: Circulating tumor cell
DAPI: 4′,6‐diamidino‐2‐phenylindole
ECOG: Eastern Cooperative Oncology Group
EMT: Epithelial–mesenchymal transition
EpCAM: Epithelial cell adhesion molecule
FDA: Food and Drug Administration
HR: Hazard ratio
IQR: Interquartile range
mCRC: Metastatic colorectal cancer
OS: Overall survival
PE: Phycoerythrin
PFS: Progression‐free survival
tdEV: Tumor‐derived extracellular vesicles

## 1 Introduction

Colorectal cancer (CRC) is one of the leading causes of cancer‐related mortality worldwide. Despite advances in surgical techniques, chemotherapy, and targeted therapies, metastatic CRC (mCRC) presents significant challenges in clinical management due to its heterogeneity and propensity to develop resistance to treatment [1]. The prognosis for mCRC patients remains poor, with only a small proportion achieving long‐term survival, highlighting the need for improved early detection, monitoring of disease progression, and personalized treatment strategies [2]. As such, identifying reliable biomarkers to assess tumor burden, predict therapeutic response, and monitor treatment efficacy is crucial in improving patient outcomes [3].

Liquid biopsies, which enable the non‐invasive detection of tumor‐derived materials, have emerged as an attractive tool in oncology. Among the various components of liquid biopsies, circulating tumor cells (CTC) have gained considerable attention due to their potential to reflect the molecular characteristics of metastatic tumors [4]. CTC are cancer cells that detach from the primary tumor and enter the bloodstream, serving as a source of real‐time information regarding the metastatic process. The enumeration and characterization of CTC offer important insights into disease status and prognosis in various cancers. In mCRC, several studies demonstrated that the presence of ≥ 3 CTC/7.5 mL blood is significantly associated with worse progression‐free survival (PFS) and overall survival (OS), supporting their clinical relevance as prognostic biomarkers [5]. Furthermore, dynamic monitoring of CTC count during treatment has been proposed as a surrogate indicator of therapeutic efficacy, potentially guiding treatment decisions and improving patient stratification [6]. However, despite the recognized prognostic value of CTC, their integration into routine clinical management of mCRC is still limited, largely due to technical challenges in their detection, enumeration, and molecular characterization [7].

A promising area of research in cancer diagnostics is represented by non‐canonical CTC harboring hematopoietic and epithelial/tumor properties, known as circulating hybrid cells (CHC) or dual‐positive cells [8, 9]. These cells, often more frequent than canonical CTC, arise from the fusion of tumor cells with immune cells, resulting in a unique population that possesses both epithelial and leukocyte characteristics. This phenomenon of cell fusion is thought to enhance the migratory and metastatic potential of tumor cells, making hybrid CTC valuable biomarkers for prognosis, treatment response, and metastasis risk [8, 9, 10]. Although the significance of CTC has been extensively documented, the specific role of CHC, particularly in mCRC, remains an uncharted field of study yet.

To date, the gold standard in CTC detection is the CellSearch^®^ system, the only FDA‐approved platform for the CTC enumeration in metastatic breast, colorectal, and prostate cancers. Despite its established clinical value, the system faces several limitations [11]. One significant drawback is its inability to detect CTC undergoing epithelial‐mesenchymal transition (EMT). Cells in EMT lose epithelial features such as the expression of epithelial cell adhesion molecule (EpCAM) or cytokeratins (CK), two critical targets in the CellSearch^®^ isolation protocol [12]. This limitation restricts the detection of a potentially important subset of CTC, which may play a crucial role in metastasis and tumor progression [13]. Additionally, cells that fall outside the canonical definition of CTC as per the CellSearch^®^ system criteria are not identified, potentially omitting other clinically relevant cell types from analysis, such as CHC [11]. The reliability of the system is further constrained by its dependence on operator expertise and visual enumeration, which can introduce variability in CTC quantification and affect reproducibility [14]. To address this latter shortcoming, the ACCEPT (Automated CTC Classification Enumeration and PhenoTyping) software was developed [15]. It offers an automated approach to the enumeration and analysis of CTC, which may improve the precision and reliability of CTC detection, particularly in mCRC patients. This system has the potential to overcome challenges associated with manual counting and subjective interpretation, offering a more standardized and efficient tool for clinical use [16].

We conducted a retrospective analysis of archived CellSearch^®^ images from mCRC patients treated at Policlinico Umberto I in Rome between 2010 and 2013. The first aim was to determine whether automated CTC selection provides a more reliable and accurate assessment by avoiding the operator‐dependent variability inherent in manual interpretation. To this end, we analyzed survival outcomes based on two CTC cutoff values: the standard threshold of ≥ 3 CTC [17] and an alternative one of ≥ 1 CTC suggested by our group [7, 18]. We also investigated whether the presence of CHC at baseline, quantified using both manual and automated analysis, might predict patient outcomes. Finally, we explored whether CHC load, alongside CTC count, might further improve risk stratification in mCRC patients.

## 2 Materials and methods

### 2.1 Study population

We retrospectively analyzed archived images generated by the CellSearch^®^ system from a cohort of 67 patients with a histopathological diagnosis of mCRC, recruited between 2010 and 2013 at Policlinico Umberto I of Rome. The inclusion criteria were as follows: male or female patients aged over 18 years, baseline CTC count, availability of archived CellSearch^®^ images, and complete clinical data. Blood samples for CTC enumeration using the CellSearch^®^ System (Menarini Silicon Biosystems, Bologna, Italy) were collected at baseline, prior to the initiation of any systemic treatment, regardless of the planned therapeutic line. A total of 7.5 mL of blood was drawn from each patient and collected into CellSave Preservative Tubes (Menarini Silicon Biosystems). Samples were stored at room temperature and processed within 72 h. Patients provided written informed consent before participating in the study, which was conducted in accordance with a protocol approved by the Institutional Review Board of Policlinico Umberto I of Rome (protocol no. 668/09, 9 July 2009; amended protocol 179/16, 1 March 2016). All patient information was managed in compliance with the ethical standards outlined in the Declaration of Helsinki.

### 2.2 Manual enumeration of circulating tumor cells and circulating hybrid cells

CTC and CHC were enumerated using the CellSearch^®^ Circulating Tumor Cells Kit (Menarini Silicon Biosystems), as previously described [7]. Briefly, after immunomagnetic enrichment, EpCAM‐positive cells were stained with specific markers to identify cellular components and distinguish cell types. Phycoerythrin‐conjugated (PE) antibodies were used to target CK 8, 18, and 19, intermediate filament proteins indicative of epithelial origin. Allophycocyanin‐conjugated (APC) antibodies labeled CD45, the leukocyte common antigen, for identifying hematopoietic cells. The nuclear dye 4',6‐diamidino‐2‐phenylindole (DAPI) was used to visualize nuclei, ensuring the detection of intact cells. After staining, samples were loaded into cartridges, and fluorescence images were captured using the semi‐automated fluorescence microscope. Built‐in software automatically identified cells positive for DNA and CK [19]. Preselected objects were displayed in an image gallery, where trained operators manually reviewed and confirmed the identity of target cells. An object was classified as a CTC if it had a diameter > 4 μm, a visible nucleus, positive staining for CK, and negative staining for CD45. Conversely, an object was classified as a CHC if it had a diameter > 4 μm, a visible nucleus, and positive staining for both CK and CD45.

### 2.3 Automatic enumeration of circulating tumor cells and circulating hybrid cells

Archived fluorescence images were re‐analyzed using ACCEPT (https://github.com/LeonieZ/ACCEPT), an open‐source tool designed for automated detection and classification of fluorescent objects, using the Full Detection function. The software processes images captured across multiple fluorescence channels (DAPI, CK, CD45) and extracts morphological and fluorescence signal intensity parameters, including mean and maximum intensities, object size, shape (eccentricity, perimeter‐to‐area ratio), and signal overlap between channels, as described in detail elsewhere [15, 20]. CTC were identified based on established gating criteria, while CHC were counted by applying leukocyte selection criteria to the CD45 signal in combination with CTC parameters [21, 22]. An object was identified to be a CTC when meeting the following criteria: mean intensity CD45 ≤ 5 arbitrary units (AU), mean intensity DAPI > 45 AU, mean intensity CK > 60 AU, CK size between 16 μm^2^ and 400 μm^2^, and CK overlay with DAPI > 0.2 AU. An object was identified to be a CHC by applying gating parameters as follows: mean CD45 intensity > 30 AU, maximum CD45 intensity > 50 AU, mean DAPI intensity > 45 AU, mean CK intensity > 60 AU, CK size between 16 μm^2^ and 400 μm^2^, CK overlay with DAPI > 0.2 AU, CD45 overlay with DAPI > 0.2 AU, and CD45 size between 16 μm^2^ and 400 μm^2^. The applied parameters are detailed in Table 1.

**Table 1:** ACCEPT gates applied for the automated enumeration of CTC and CHC. AU, arbitrary units; CHC, circulating hybrid cells; CK, cytokeratin; CTC, circulating tumor cells; DAPI, 4',6‐diamidino‐2‐phenylindole; FITC, fluorescein isothiocyanate; PE, phycoerythrin.

Parameters	CTC	CHC
Mean intensity CD45	≤ 5 AU	> 30 AU
Max intensity CD45	—	> 50 AU
Mean intensity DAPI	>45 AU	> 45 AU
Mean intensity CK	>60 AU	> 60 AU
Max intensity CK	—	—
Area CK	—	—
Eccentricity CK	—	—
Perimeter to area CK	—	—
Perimeter CK	—	—
CK size	> 16 μm^2^	> 16 μm^2^
CK size	≤ 400 μm^2^	≤ 400 μm^2^
CK overlay with DAPI	> 0.2 AU	> 0.2 AU
Mean intensity marker 1 (PE)	≤ 5 AU	≤ 5 AU
Mean intensity marker 2 (FITC)	≤ 5 AU	≤ 5 AU
CD45 overlay with DAPI	—	> 0.2 AU
CD45 size	—	> 16 μm^2^
CD45 size	—	≤ 400 μm^2^

### 2.4 Statistical analysis

Continuous variables were summarized as medians with interquartile ranges (IQR), while categorical variables were expressed as counts and percentages. Spearman's rank correlation coefficient was used to assess relationships between CTC and CHC counts, as well as between PFS and OS. PFS was defined as the time from treatment initiation to disease progression or last follow‐up without progression, whereas OS was measured from the start of first‐line treatment to death from any cause or last follow‐up. The non‐parametric Spearman rank correlation test was applied to evaluate associations between CHC/CTC counts and OS/PFS. The non‐parametric Mann–Whitney *U* test and Kruskal–Wallis test were used to assess associations between CHC counts (measured using CellSearch^®^ and ACCEPT) and clinical variables such as sex, RAS status (mutated vs. wild‐type), metastasis, treatment line, tumor sidedness, grade, and regional node involvement. OS was estimated using Kaplan–Meier survival curves, and differences between groups were assessed via the log‐rank test. Cox proportional hazards regression models, both univariate and multivariate, were applied to determine the effect of double positivity (concurrent detection of CTC and CHC), CTC, and CHC on survival outcomes, reporting hazard ratios (HR) with 95% confidence intervals (CI). Fisher's exact test was used to assess the classification accuracy of discordant patients compared to Kaplan–Meier models based on concordant cases. Statistical significance was set at *P* ≤ 0.05, and analyses were performed using R Statistical Software (version 4.4.2; R Core Team 2024).

## 3 Results

### 3.1 Patients and data collection

The study population comprised 67 patients with mCRC, with a median age of 62.5 years (IQR: 55–69.5 years). Of these, 44 patients (65.67%) were male, and 23 (34.33%) were female. Regarding tumor location, 17 patients (25.4%) had rectal cancer, with the remaining cases distributed between right‐ (*n* = 24, 35.8%) and left‐sided (*n* = 26, 38.8%) colon cancers. Tumor grading, assessed according to standard histopathological criteria, revealed 1 patient (1.5%) with G1, 49 (73.1%) with G2, and 17 (25.4%) with G3 tumors. RAS mutational status, assessed on tumor tissue, identified 28 wild‐type cases (41.8%) and 39 ones harboring RAS mutations (58.2%). Metastatic involvement was observed in the liver (*n* = 47, 70.1%), lung (*n* = 33, 49.3%), peritoneum (*n* = 12, 17.9%), and bone (*n* = 4, 6%). Multiple metastases were observed in 24 patients (35.8%), while 43 patients (64.2%) had single‐site metastases. Lymph node involvement was categorized as N0 (*n* = 28, 41.8%), N1 (*n* = 21, 31.3%), and N2 (*n* = 18, 26.9%). Performance status, evaluated using the Eastern Cooperative Oncology Group (ECOG) scale, showed 46 patients (68.7%) scoring 0, 15 patients (22.4%) scoring 1, and 6 patients (8.9%) scoring 2. Of the patients, 45 (67.16%) underwent first‐line, 12 (17.91%) second‐line, 6 (8.96%) third‐line, and 4 (5.97%) fourth‐line treatment. The clinicopathological characteristics of the patients at baseline are summarized in Table 2 and detailed in Table S1.

**Table 2:** Baseline characteristics of the study population (*N* = 67). IQR, interquartile range; yrs, years.

Characteristics	No. (%)
**Age (yrs)**	
Median age (IQR)	62.5 (55–69.5)
**Sex**	
F	23 (34.33%)
M	44 (65.67%)
**Tumor Sidedness**	
Left	26 (38.8%)
Right	24 (35.8%)
Rectum	17 (25.4%)
**Stage**	
IV	67 (100%)
**RAS status (tumor tissue)**	
Wild‐type	28 (41.8%)
Mutated	39 (58.2%)
**Grade**	
G1	1 (1.5%)
G2	49 (73.1%)
G3	17 (25.4%)
**Metastasis**	
Single site	43 (64.2%)
Multiple sites	24 (35.8%)
**Metastatic site**	
Liver	47 (70.1%)
Lung	33 (49.3%)
Peritoneum	12 (17.9%)
Bone	4 (6%)
**Regional node involvement**	
N0	28 (41.8%)
N1	21 (31.1%)
N2	18 (26.9%)
**Performance status**	
0	46 (68.7%)
1	15 (22.4%)
2	6 (8.9%)
**Line of therapy**	
1st	45 (67.16%)
2nd	12 (17.91%)
3rd	6 (8.96%)
4th	4 (5.97%)

### 3.2 Manual and automated enumeration of CTC and CHC

CTC and CHC were enumerated from stored digital images using both the manual and automated approaches. Manual enumeration identified a total of 1103 CTC in 30 out of 67 patients (44.8%) and 92 CHC in 38 out of 67 patients (56.7%). The automated method detected 1001 CTC and 74 CHC in 37 out of 67 patients (55.2%) and 35 out of 67 patients (52.2%), respectively. The mean CTC count was 16.5 for manual enumeration and 14.9 for the automated one. Median CTC counts were 0 (IQR: 0–1) for manual enumeration and 1 (IQR: 0–2) for automated analysis. Median CHC counts were 1 (IQR: 0–2) for manual enumeration and 1 (IQR: 0–1) for automated enumeration, with mean CHC values of 1.4 and 1.1, respectively. Manual enumeration achieved higher CTC detection rates than ACCEPT in 9 cases (13.4%). However, ACCEPT uniquely detected CTC in 13 (19.4%) cases, compared to 6 (9%) found to be CTC‐positive exclusively through the manual approach. The overall concordance rate for CTC detection between the two methods was 71.6%, with 24 patients (35.8%) showing no CTC and 24 ones (35.8%) having at least one CTC. Among these, numerical discordance was observed in 19 cases (28.4%). Additionally, 12 individuals had ≥ 3 CTC detected by both systems, with a concordance rate of 75%. Representative examples of concordance and discordance between manual and automated classification of CTC are shown in Fig. S1. For CHC detection, the concordance rate between methods was 58.2%. Specifically, 17 cases (25.4%) were CHC‐negative and 22 (32.8%) were CHC‐positive with both methods. Numerical concordance was observed in 8 cases (11.9%) with 1 CHC, 1 case (1.5%) with 2 CHC, and 1 case (1.5%) with 4 CHC detected by both methods. Representative CHC, including examples of concordance and discordance between manual and automated classification, are presented in Fig. S2. All CTC and CHC counts obtained using both methods are reported in Table S1.

### 3.3 Correlative and comparative analysis of manual and automated CTC enumeration for survival prognosis

The correlation analysis between enumeration methods showed a moderate correlation between CTC counts detected by CellSearch^®^ (CTC^manual^) and ACCEPT (CTC^ACCEPT^) (Spearman's *ρ* = 0.59). In terms of clinical outcomes, the median OS was 32 months (IQR: 21–62.5), while the median PFS was 12 months (IQR: 6–19). A strong correlation between OS and PFS (Spearman's *ρ* = 0.76) suggests that both measures reflect the same underlying disease progression, supporting the robustness of OS as a clinical outcome. To further investigate the prognostic value of CTC counts, we analyzed their correlation with OS and PFS. A moderate negative correlation was found between CTC^manual^ and OS (Spearman's *ρ* = −0.41, *P* < 0.001), as well as between CTC^ACCEPT^ and OS (Spearman's *ρ* = −0.32, *P* < 0.001). Similarly, CTC counts negatively correlated with PFS, with Spearman's *ρ* values of −0.45 (*P* < 0.001) for CTC^manual^ and −0.42 (*P* < 0.001) for CTC^ACCEPT^. These findings reinforce the role of CTC enumeration as a prognostic marker, with higher CTC counts associated with poorer survival outcomes.

**Fig. 1:**
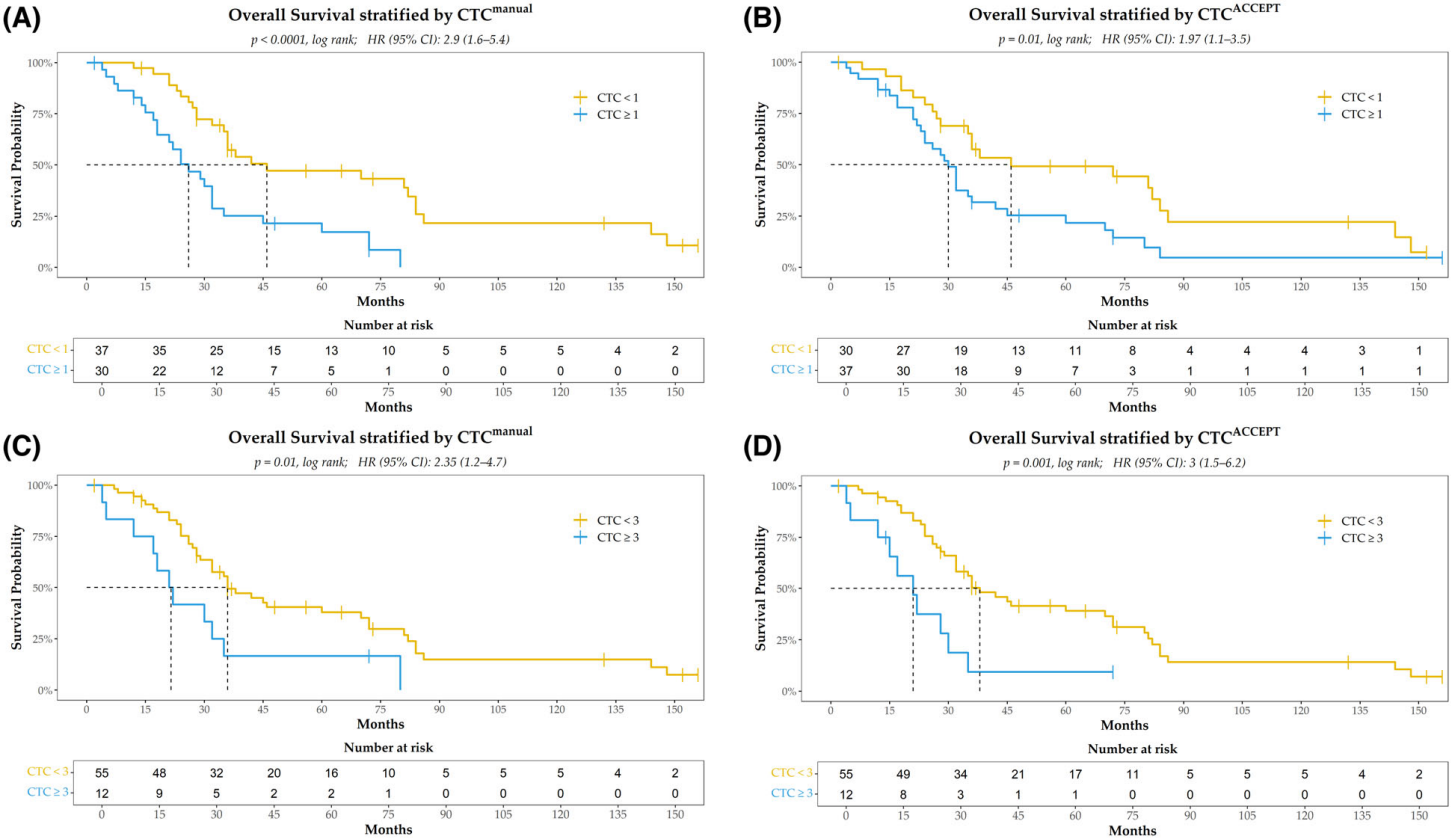
Kaplan–Meier survival curves for patients (*N* = 67) stratified by circulating tumor cell (CTC) count using manual and automated methods. Overall survival stratified by manual (CellSearch^®^) (A) and automated (ACCEPT) (B) CTC enumeration of patients divided into two groups based on the presence of ≥ 1 CTC. Overall survival stratified by manual (C) and automated (D) CTC enumeration of patients divided into two groups based on the presence of ≥ 3 CTC. For all panels, the log‐rank test was used to assess the differences in survival between groups. Hazard ratios (HR) and 95% confidence intervals (CI) are provided for each comparison. The tick marks indicate censored data.

Further analysis of survival outcomes based on CTC counts obtained through manual vs. automated enumeration confirmed the prognostic significance of both methods, albeit with differing sensitivity. Using a cutoff of ≥ 1 CTC, manual enumeration showed a median OS of 46 months (95% CI: 4.67–87.32) for patients with < 1 CTC, compared to a markedly shorter OS of 26 months for those with ≥ 1 CTC (*P* < 0.0001; HR 2.9, 95% CI: 1.6–5.4) (Fig. 1A). A similar trend was observed with ACCEPT‐based enumeration, where the median OS was 46 months for patients with < 1 CTC and 30 months for those with ≥ 1 CTC (*P* = 0.01; HR 1.97, 95% CI: 1.1–3.5) (Fig. 1B). While both methods identified a significant difference in survival, the higher hazard ratio observed with manual enumeration (HR 2.9 vs. 1.97) suggests it may be more sensitive in distinguishing high‐risk patients at this threshold.

Further stratification using a ≥ 3 CTC cutoff confirmed the prognostic value of both methods. With manual enumeration, patients with < 3 CTC had a median OS of 36 months, significantly longer than the 21 months observed in those with ≥ 3 CTC (*P* = 0.01; HR 2.35, 95% CI: 1.2–4.7) (Fig. 1C). A similar pattern was observed with automated enumeration, where patients with < 3 CTC had a median OS of 38 months, whereas those with ≥ 3 CTC had a significantly shorter OS of 21 months (*P* = 0.001; HR 3, 95% CI: 1.5–6.2) (Fig. 1C). Notably, at the ≥ 3 CTC threshold, automated enumeration yielded a higher hazard ratio (3 vs. 2.35), suggesting it may more precisely identify high‐risk patients at this level. However, this increased sensitivity at the ≥ 3 CTC cutoff might come at the cost of misclassifying patients with 1–2 CTC as lower risk, potentially explaining the reduced OS observed in the automated method at the ≥ 1 CTC threshold.

### 3.4 ACCEPT software improves CTC enumeration in mCRC

**Fig. 2:**
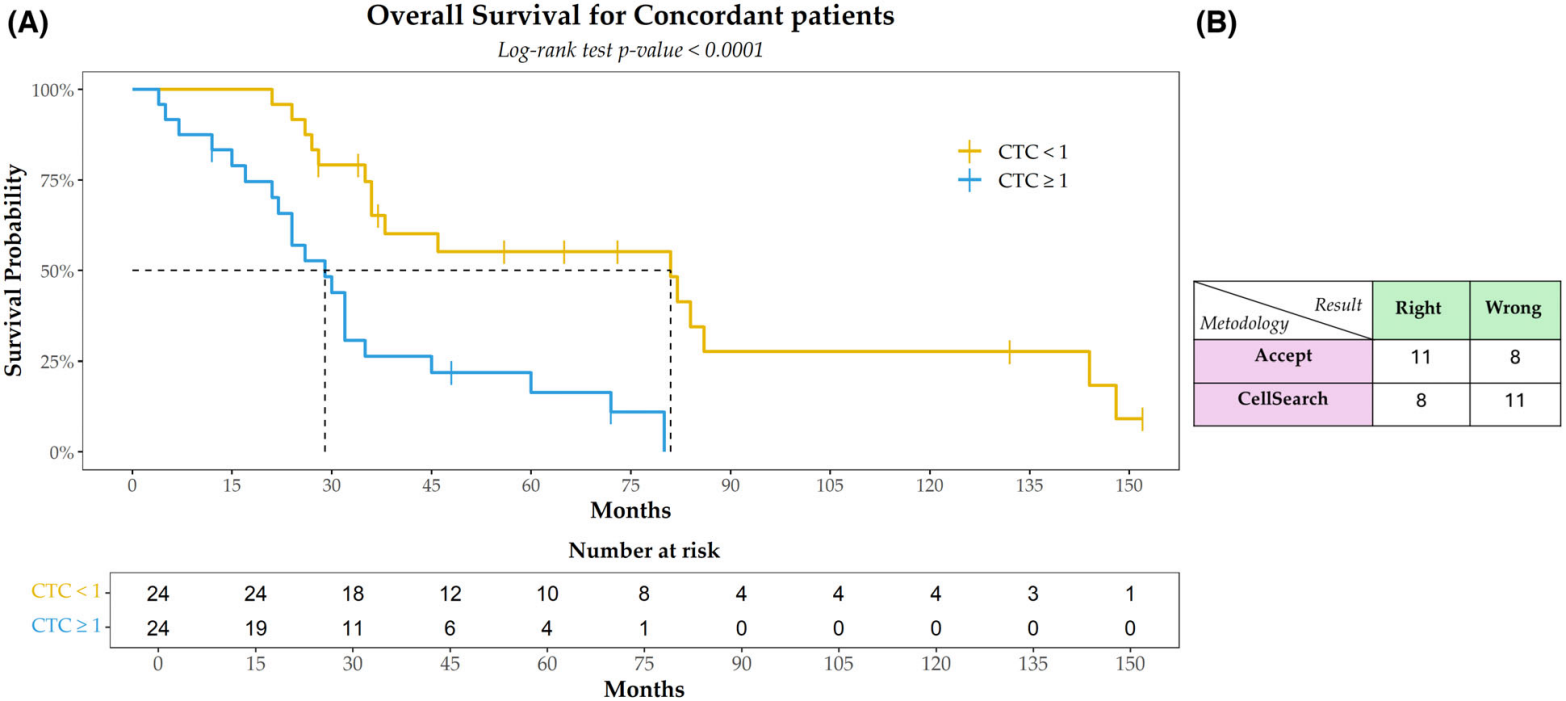
Survival analysis at the cutoff of ≥ 1CTC. Kaplan–Meier plot for overall survival (OS) in the cohort of concordant patients (*N* = 48) identified using a cutoff of ≥ 1 circulating tumor cell (CTC). For each group, the median OS value (in months) and the number of patients at different time points are presented. The log‐rank test *P*‐value is shown. The dashed line indicates the median survival time in each group (A). 2 × 2 classification matrix displaying the number of patients (*N* = 19) with concordant and discordant results between the two methods (B). The tick marks indicate censored data.

In order to determine whether CTC counting with the ACCEPT software improves reliability and accuracy compared to the manual method, we analyzed survival outcomes based on two predefined cutoff values: ≥ 1 CTC and ≥ 3 CTC. Concordant patients, defined as those with consistent CTC counts across both methods, were used as a benchmark cohort to construct Kaplan–Meier survival models. The survival expectations of discordant patients were then assessed relative to these models to determine which method better aligned with actual clinical outcomes. The Kaplan–Meier analysis for patients with concordance at a cutoff of ≥ 1 CTC revealed a significant difference in OS. Patients classified as negative (0 CTC) had a median OS of 81 months, whereas those classified as positive (≥ 1 CTC) had a median OS of 26 months (*P* < 0.0001, log‐rank test), demonstrating a clear prognostic value of CTC enumeration at this threshold (Fig. 2A). Among the 19 discordant patients (28.3% of the cohort), a 2 × 2 classification matrix was constructed to evaluate the accuracy of the two methodologies. The results showed that ACCEPT correctly classified 11 out of 19 patients (57.9%), while CellSearch^®^ correctly classified 8 out of 19 patients (42.1%) (Fig. 2B). However, the difference between the two methods was not statistically significant (*P* = 0.5, Fisher's exact test).

**Fig. 3:**
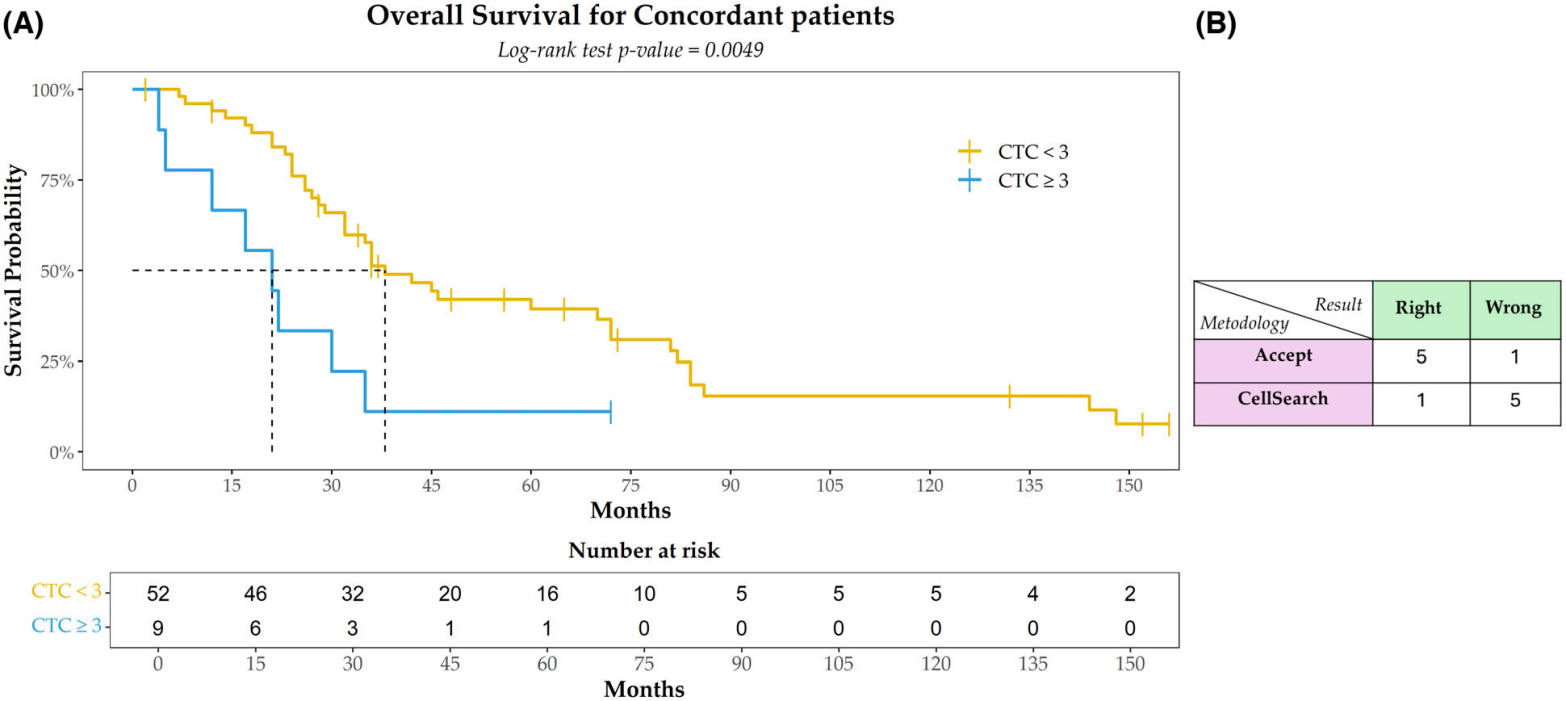
Survival analysis at cutoff of ≥ 3 CTC. Kaplan–Meier plot for overall survival (OS) in the cohort of concordant patients (*N* = 61) identified using a cutoff of ≥ 3 circulating tumor cells (CTC). For each group, the median OS value (in months) and the number of patients at different time points are presented. The log‐rank test *P*‐value is shown. The dashed line indicates the median survival time in each group (A). 2 × 2 classification matrix displaying the number of patients (*N* = 6) with concordant and discordant results between the two methods (B). The tick marks indicate censored data.

At the ≥ 3 CTC cutoff, the Kaplan–Meier model again indicated a statistically significant difference in survival. Patients classified as negative (≤ 2 CTC) had a median OS of 38 months, whereas those classified as positive (≥ 3 CTC) had a median OS of 21 months (*P* = 0.0049, log‐rank test). It is worth noting that the sample sizes for these groups were highly unbalanced, with a greater number of negative patients (*N* = 52) compared to positive patients (*N* = 9) (Fig. 3A). For the 6 discordant patients (8.9% of the cohort), the classification matrix indicated that ACCEPT correctly classified 5 out of 6 patients (83.3%), while CellSearch^®^ correctly classified only 1 out of 6 patients (16.7%) (Fig. 3B). Although the *P*‐value (*P* = 0.08, Fisher's exact test) was not statistically significant, the observed trend suggests that a larger sample size may lead to a more definitive conclusion regarding the superior accuracy of ACCEPT in classifying CTC.

### 3.5 Prognostic value of CHC and their correlation with CTC

**Fig. 4:**
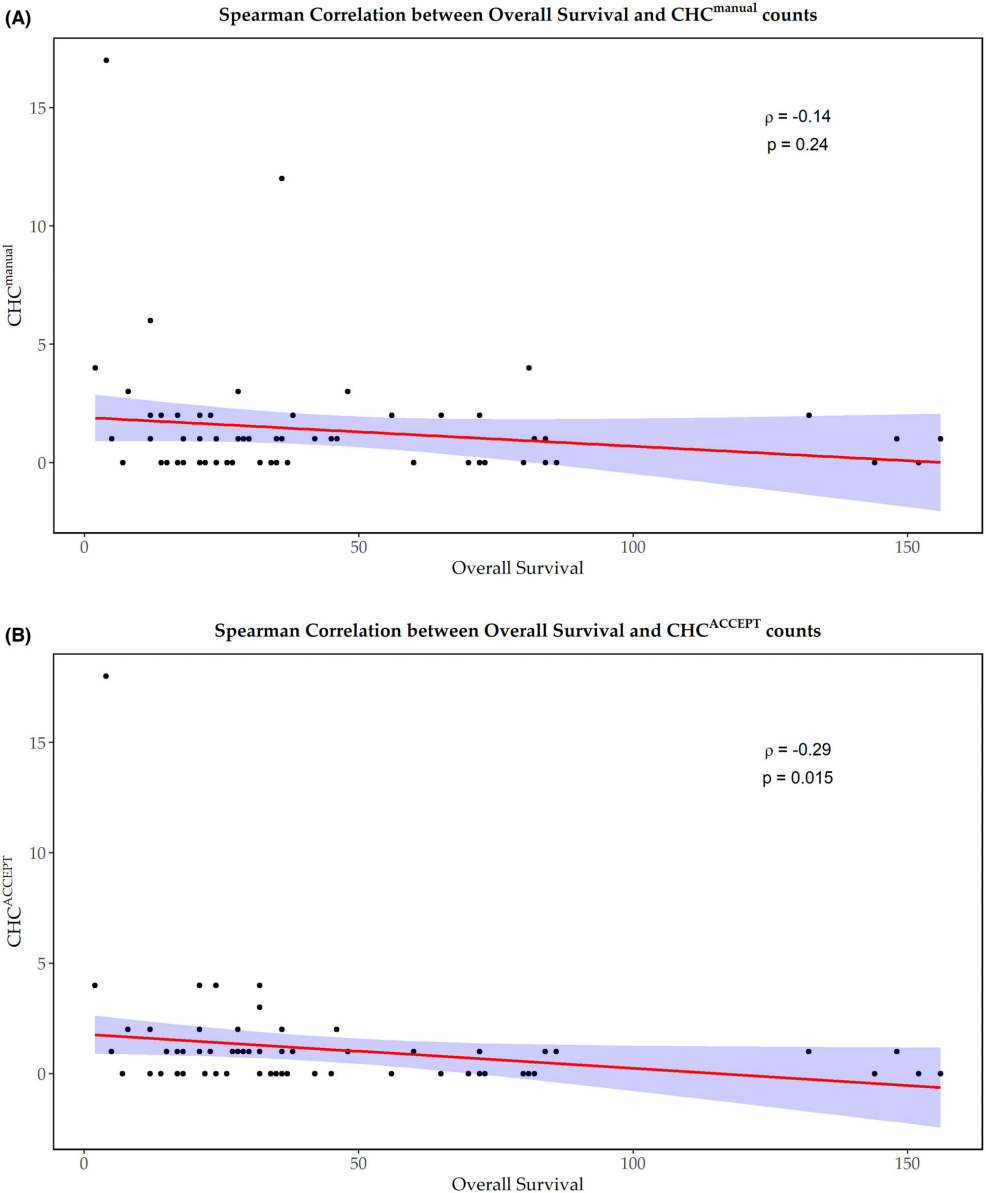
Spearman correlation between overall survival and circulating hybrid cell (CHC) counts. Spearman correlation analysis was performed to assess the relationship between overall survival and a) CHC counts by CellSearch^®^ (CHC^manual^) and b) CHC counts using ACCEPT (CHC^ACCEPT^). A 95% confidence interval (CI) was used to evaluate the results.

The analysis of correlations between enumeration methodologies revealed a weak correlation between CHC^manual^ and CHC^ACCEPT^ (Spearman's *ρ* = 0.2). No substantial correlation was found between CHC and CTC both manually enumerated (Spearman's *ρ* = −0.04) or between CHC^ACCEPT^ and CTC^manual^ (Spearman's *ρ* = 0.11). Similarly, CHC^manual^ and CTC^ACCEPT^ showed a weak inverse correlation (Spearman's *ρ* = −0.08), while CHC and CTC enumerated using the ACCEPT method displayed a low positive correlation (Spearman's *ρ* = 0.14). We then investigated the relationship between CHC counts and clinical outcomes, specifically OS and PFS. A low negative correlation was found between CHC^manual^ and OS (Spearman's *ρ* = −0.14, *P* = 0.24) and between CHC^ACCEPT^ and OS (Spearman's *ρ* = −0.28, *P* = 0.01) (Fig. 4). Regarding PFS, similar negative correlations were observed with CHC^manual^ (Spearman's *ρ* = −0.28, *P* = 0.01) and CHC^ACCEPT^ (Spearman's *ρ* = −0.25, *P* = 0.03). These findings indicate that CHC exhibits weaker correlations across the two enumeration methods compared to CTC. Except for the association between CHC^manual^ and OS, all other relationships reached statistical significance, though to a lesser extent than those observed for CTC, suggesting that the prognostic impact of CHC may be lower or more complex to interpret. The prognostic significance of CHC was further assessed through statistical tests on various clinical parameters. For manual CHC counts, the Mann–Whitney *U* test showed no significant association with sex (*P* = 0.97), RAS mutation status (*P* = 0.18), or metastatic site (*P* = 0.67). Similarly, the Kruskal–Wallis test revealed no significant differences for therapy line (*P* = 0.41), tumor sidedness (*P* = 0.12), tumor grade (*P* = 0.43), or regional lymph node involvement (*P* = 0.37). For automated CHC counts, no significant associations were found for sex (*P* = 0.12), RAS status (*P* = 0.09), metastatic site (*P* = 0.87), therapy line (*P* = 0.94), tumor sidedness (*P* = 0.29), tumor grade (*P* = 0.62), or regional lymph node involvement (*P* = 0.52). The lack of significance aligns with the low correlation observed between the two enumeration methods (Spearman's *ρ* = 0.2).

To explore whether the use of different cutoff values could improve patient stratification, we followed the procedure described in Section 3.3. However, the comparative analysis of survival outcomes confirmed that CHC counts cannot be considered independent prognostic factors for OS. Using a cutoff of ≥ 1 CHC, manual enumeration showed a median OS of 36 months for patients with < 1 CHC compared to 32 months for those with ≥ 1 CHC (*P* = 0.93; HR 0.9, 95% CI: 0.5–1.7) (Fig. S3a). The ACCEPT‐based enumeration yielded similar results, with a median OS of 42 months for patients with < 1 CHC vs. 30 months for those with ≥ 1 CHC (*P* = 0.15; HR 1.5, 95% CI: 0.9–2.6) (Fig. S3b). Although neither method produced statistically significant results, the survival model built on CHC^ACCEPT^ counts appeared to yield more intriguing trends.

Additionally, we evaluated a cutoff of ≥ 3 CHC to determine whether a different threshold could lead to distinct findings. With the manual enumeration method, patients with < 3 CHC had a median OS of 35 months, compared to 28 months in those with ≥ 3 CHC (*P* = 0.26; HR 1.62, 95% CI: 0.7–3.8) (Fig. S3c). For automated enumeration, patients with < 3 CHC exhibited a median OS of 36 months, while those with ≥ 3 CHC had a significantly shorter median OS of 24 months (*P* = 0.002; HR 3, 95% CI: 1.2–8.1) (Fig. S3c). Despite its statistical significance, this result was likely influenced by bias due to the imbalance in patient numbers between groups (*n* = 61 vs. *n* = 6).

## 4 Discussion

The enumeration of CTC using the CellSearch^®^ system in mCRC presents unique challenges compared to other malignancies such as breast and prostate cancer, where CTC counts have demonstrated strong prognostic significance [23, 24]. In mCRC, studies have consistently reported lower CTC detection rates, with only 30–40% of patients exhibiting CTC levels above the conventional prognostic threshold of ≥ 3 CTC per 7.5 mL of blood, a cutoff that has shown limitations in stratifying patients into meaningful prognostic groups [7, 18, 25]. This low detection rate raises concerns about the sensitivity of enumeration methods and potential operator‐dependent variability, which can introduce inconsistencies in CTC classification [16, 26]. Such challenges emphasize the need for improved methods to enhance detection reliability and prognostic assessment.

To address this issue, we evaluated whether the use of the ACCEPT software could enhance CTC detection rates and improve prognostic accuracy compared to the standard manual approach. Our findings revealed a moderate correlation between manual CTC enumeration using CellSearch^®^ and automated enumeration via ACCEPT, indicating that while the two methods provide comparable results, they are not entirely interchangeable. Unlike mCRC, a strong correlation between manual and ACCEPT‐based enumeration has been observed in metastatic breast cancer, with discrepancies mainly occurring at low CTC counts, particularly below the 5 CTC/7.5 mL threshold [27]. A similar trend was reported in metastatic castration‐resistant prostate cancer, where a linear relationship between the two methods was observed at low CTC levels (≤ 5 CTC/7.5 mL), but with increasing variability at higher counts [28]. These discrepancies highlight the tumor‐specific challenges in colorectal cancer, which may be attributed to distinct tumor biology and different mechanisms of hematogenous dissemination compared to breast and prostate cancers. Notably, tumor localization plays a significant role in CTC characteristics, with right‐ and left‐sided colorectal cancers showing distinct molecular profiles, metastatic patterns, and interactions with the tumor microenvironment that affect CTC detection [29]. Additionally, colorectal CTC tend to be smaller and morphologically similar to leukocytes, making manual identification more challenging, especially when CTC are interspersed among white blood cells [30]. This increases the likelihood of underestimation in manual counts, while the ACCEPT software, which uses multiple morphometric parameters, can compensate for these limitations and enhance CTC classification accuracy in colorectal cancer [30]. Our cohort revealed that manual enumeration detected CTC in 44.8% of patients, whereas ACCEPT identified them in 55.2%, with numerical discrepancies occurring in 28.4% of cases. This suggests that the automated method improves sensitivity, particularly in detecting low CTC counts, but introduces variability near clinically relevant thresholds. The survival analysis at different CTC cutoff values revealed discrepancies between manual counting and ACCEPT software‐based quantification. Specifically, at a threshold of ≥ 1 CTC, manual enumeration yielded a higher hazard ratio compared to ACCEPT, although both methods identified significant prognostic differences at this level. This finding confirms our previous observations [7, 18]. In 2013, we demonstrated that the presence of at least one CTC is associated with worse prognosis, supporting its role as a clinically relevant threshold for risk stratification [18]. More recently, our work on mCRC reinforced the prognostic significance of this cutoff, showing that even minimal CTC presence correlates with disease progression and OS [7]. However, our current findings reveal that at the ≥ 3 CTC cutoff, the trend reverses, with ACCEPT showing a higher hazard ratio than manual enumeration, suggesting improved performance in identifying high‐risk patients at this level. These findings indicate that the enumeration technique chosen significantly influences prognostic stratification. A potential explanation for these differences lies in the sensitivity and specificity of each method. Manual counting may provide higher sensitivity at low CTC numbers, as human observation can recognize subtle morphological features that automated algorithms might overlook [31]. This may lead to an increased HR at the ≥ 1 CTC threshold, suggesting that even a minimal presence of CTC, when detected manually, has a significant prognostic impact. However, manual counting is subject to inter‐operator variability, which could introduce inconsistencies, particularly at higher CTC burdens [15]. In contrast, the automated method benefits from advanced image processing algorithms that enhance its ability to identify subtle morphological distinctions, such as cell size, shape, and granularity, which can be difficult to assess consistently in manual enumeration [15, 20]. This capability becomes particularly advantageous at higher CTC counts, where ACCEPT standardized criteria for CTC identification reduce false positives and increase specificity. The improved sensitivity and specificity observed with ACCEPT at the ≥ 3 CTC cutoff allow for a more effective discrimination between high‐ and low‐risk patients. This finding aligns with previous studies that have demonstrated the efficacy of automated classifiers at higher thresholds. While the automated system may face challenges at lower CTC counts, its performance significantly improves as the threshold increases, providing more accurate prognostic stratification of patients with higher CTC levels [27]. These observations highlight the importance of standardization and the potential of the automated approach to capture a biologically relevant subset of CTC, which are more strongly associated with patient prognosis. Our findings indicate that manual and automated CTC enumeration methods may serve complementary roles. While manual counting appears advantageous for early risk assessment due to its higher sensitivity at lower CTC numbers, ACCEPT provides a more robust and reproducible prognostic stratification at higher CTC counts. This dual approach suggests that integrating both methods might optimize patient classification, improving risk assessment and potential therapeutic decision‐making. Future studies should further explore these differences in larger patient cohorts and investigate strategies to standardize manual counting while refining the automated approach to enhance its sensitivity.

Beyond CTC enumeration, we also investigated whether the presence of CHC at baseline might have a prognostic role, either alone or alongside CTC count, to further refine risk stratification in mCRC patients. CHC, defined by the co‐expression of epithelial (CK/EpCAM) and hematopoietic (CD45) markers, was enumerated using both manual and automated methods [32]. The concordance rate between these approaches was low (58.2%), with a weak correlation (Spearman's *ρ* = 0.2), reflecting the phenotypic and morphological heterogeneity of CHC and the inherent variability in detection methods. Automated enumeration, performed using the ACCEPT software, demonstrated potential for reducing operator‐dependent variability by applying objective gating parameters based on fluorescence intensity and morphological features [21, 22]. Despite these advantages, CHC counts remained low across both enumeration methods, with a median of 1 CHC per patient. This suggests that CHC is less frequent in mCRC compared to other malignancies, such as breast and pancreatic cancers, where they are more abundant and show stronger prognostic associations [8, 9, 33]. However, direct comparative studies across cancer types are needed to confirm this trend.

Regarding their prognostic significance, we observed weak negative correlations between CHC counts and OS or PFS. Notably, automated CHC counts exhibited a slightly stronger association with OS and PFS compared to manual counts. Although these correlations reached statistical significance, their magnitude remains modest, indicating that CHC may have a limited independent prognostic role in mCRC. Furthermore, CHC counts showed no significant associations with key clinical parameters such as RAS status (mutated vs. wild‐type), metastatic site, or tumor grade. This underscores the complexity of mCRC biology and the ongoing challenge of identifying reliable biomarkers for clinical decision‐making [34, 35]. The absence of significant correlations between CHC and these clinical factors further differentiates them from CTC, which have consistently demonstrated stronger prognostic utility in mCRC [36].

The low abundance of CHC in our cohort may be partially explained by the biological and mechanistic factors influencing their circulation dynamics and potential organotropism. Unlike CTC, which can directly detach from the primary tumor and enter the bloodstream, CHC might result from heterotypic fusion events between tumor cells and immune cells, such as macrophages, that infiltrate the tumor microenvironment [37]. This fusion process, although biologically significant, may occur at a low frequency, contributing to the scarcity of CHC in circulation. Moreover, the survival and detection of CHC in the bloodstream might be influenced by their expression of specific integrins and adhesion molecules acquired through fusion, which impact their ability to interact with endothelial cells and extravasate into premetastatic niches [37]. Interestingly, the differential metastatic patterns observed in colorectal cancer could also play a role in the circulation patterns and abundance of CHC. For instance, distal colon and rectal tumors preferentially metastasize to the liver via the portal circulation, while more distal rectal cancers exhibit a higher tendency to metastasize to the lungs due to direct access to the systemic circulation [38, 39]. This differential pattern of blood flow may selectively influence the presence and distribution of CHC in circulation, depending on their capacity to traffic through specific vascular routes. In addition, the role of premetastatic niches in influencing CHC behavior cannot be overlooked. The premetastatic niche is characterized by an inflammatory microenvironment, enriched in immune cells, extracellular matrix remodeling factors, and cytokines, which can facilitate the arrest, extravasation, and colonization of CTC and hybrids [37]. Due to their hybrid phenotype, CHC may be better equipped to exploit these niches, particularly through interactions mediated by β2 integrins and other leukocyte‐specific adhesion molecules acquired during the fusion process [37]. This might confer an advantage in metastatic seeding, even if their overall presence in peripheral blood is low. Overall, the low abundance of CHC in mCRC, coupled with their limited prognostic impact in our study, may reflect their complex biology, the rarity of tumor–immune cell fusion events, and the influence of organ‐specific circulation dynamics. Future research should aim to elucidate the mechanisms underlying CHC formation, survival, and functional heterogeneity to better understand their potential role in cancer dissemination and to evaluate whether their detection can be optimized to improve prognostic accuracy in mCRC and other malignancies. While automated enumeration offers promise for improving reproducibility and sensitivity, further optimization is needed to enhance its clinical utility. Preliminary findings suggest that higher automated CHC counts are associated with poorer prognosis; however, given the weak correlations observed, these results require validation in larger cohorts with standardized methodologies. Future research should also explore integrating CHC analysis with complementary biomarkers such as circulating tumor DNA or immune profiling to refine risk stratification and therapeutic decision‐making in mCRC patients. Overall, while CHC appear less clinically actionable than CTC in mCRC, their unique biology warrants further investigation to clarify their potential role in tumor progression and immune interactions.

This study presents several limitations that should be acknowledged. First, its retrospective design and the limited number of patients may reduce the generalizability of the findings. Although the ACCEPT software provided encouraging results in improving CTC detection and prognostic stratification, our conclusions require validation in larger, prospective cohorts. Second, the lack of molecular characterization of CTC and CHC limits the possibility to explore their functional heterogeneity and predictive potential. Third, clinical data regarding microsatellite instability, DNA mismatch repair deficiency status, and specific RAS mutations were not available for detailed subgroup analyses, due to the historical timeframe of patient enrollment. Finally, the ACCEPT gating parameters used in this study were applied as established in previous publications, without specific re‐optimization for CRC [21, 22]. While this ensured methodological consistency, it might not fully account for morphological differences in CTC across tumor types, particularly their smaller size in mCRC, which could result in under‐detection or misclassification of small or atypical cells. Future studies could benefit from tumor‐specific validation and refinement of these thresholds to maximize sensitivity and accuracy.

Despite these limitations, our approach demonstrates the feasibility and potential clinical value of implementing automated CTC enumeration methods. The use of standardized, operator‐independent tools such as ACCEPT might enhance reproducibility and allow for broader application of CTC‐based prognostic assessment in routine clinical settings. Future prospective trials integrating CTC quantification with other circulating biomarkers and molecular features might facilitate the incorporation of liquid biopsy approaches into personalized treatment strategies for mCRC patients. Among these biomarkers, tumor‐derived extracellular vesicles (tdEV), which can be automatically enumerated by ACCEPT, have shown clinical relevance in several cancers and represent a valuable complement to CTC analysis in mCRC [19, 40]. In this regard, Cieslik et al. applied ACCEPT to intraoperative samples from the tumor‐draining vein and central venous catheter in CRC patients, optimizing gating parameters and performing detailed phenotypic analyses of CTC and tdEV [41]. While their work emphasizes the role of tdEV and CTC heterogeneity, our study focuses on CTC and CHC, the latter representing a still poorly characterized circulating cell population in mCRC. Together, these studies demonstrate the versatility of ACCEPT for automated assessment of distinct circulating biomarkers and its potential integration into multi‐analyte liquid biopsy frameworks.

## 5 Conclusions

This study highlights the unique challenges in enumerating CTC mCRC compared to other malignancies. The use of the ACCEPT software improved CTC detection rates, particularly at higher thresholds, offering more robust prognostic stratification compared to manual enumeration, which proved more sensitive at low CTC levels. However, both methods have complementary roles. Regarding CHC, its prognostic relevance in mCRC was limited, with low concordance between enumeration methods and weak correlations with survival. Further studies are needed to optimize enumeration methods and clarify the biological role of CHC in mCRC, possibly by integrating analysis with other biomarkers.

## Conflict of interest

The authors declare no conflict of interest.

## Author contributions

Conceptualization and methodology: MDM, PG, CN. Software and formal analysis: MS, AV. Investigation: MDM, MS, OG, CN. Resources: AZ, PG. Data curation: MDM, MS, OG, AV. Writing‐original draft preparation: AV, CN. Writing‐review and editing: MDM, PG, CN. Project administration: CN. Funding acquisition: AZ, PG. All authors have read and agreed to the published version of the manuscript.

## Supporting information


**Fig. S1.** Representative images demonstrating concordance and discordance in the classification of circulating tumor cells (CTC) by manual (CellSearch^®^) and automated (ACCEPT) analysis. Panels 1–2: CTC concordantly identified by both CellSearch^®^ and ACCEPT. Panels 3–6: Cells identified as CTC by CellSearch^®^ but not recognized by ACCEPT. Panels 7–9: Cells detected as CTC by ACCEPT but not by CellSearch^®^. Each image panel includes DAPI (nuclear stain), cytokeratin (CK‐PE), CD45 (APC), and a composite overlay for morphological assessment. APC, allophycocyanin; DAPI, 4′,6‐diamidino‐2‐phenylindole; PE, phycoerythrin.

**Fig. S2.** Representative images demonstrating concordance and discordance in the classification of circulating hybrid cells (CHC) by manual (CellSearch^®^) and automated (ACCEPT) analysis. Panels 1–4: CHC concordantly identified by both CellSearch^®^ and ACCEPT. Panel 5: CHC identified by CellSearch^®^ but not classified as CHC by ACCEPT. Panels 6–8: CHC detected by ACCEPT but not identified as CHC by CellSearch^®^. Each image panel includes DAPI (nuclear stain), cytokeratin (CK‐PE), CD45 (APC), and a composite overlay for morphological assessment. APC, allophycocyanin; DAPI, 4′,6‐diamidino‐2‐phenylindole; PE, phycoerythrin.

**Fig. S3.** Kaplan–Meier survival curves for patients (*N* = 67) stratified by circulating hybrid cell (CHC) count using manual and automated methods. Overall survival stratified by manual (CellSearch^®^) (a) and automated (ACCEPT) (b) CHC enumeration of patients divided into two groups based on the presence of ≥ 1 CHC. Overall survival stratified by manual (c) and automated (d) CHC enumeration of patients divided into two groups based on the presence of ≥ 3 CHC. For all panels, the log‐rank test was used to assess the differences in survival between groups. Hazard ratios (HR) and 95% confidence intervals (CI) are provided for each comparison. The tick marks indicate censored data.

**Table S1.** Clinical and molecular characteristics of patients at baseline: CTC and CHC counts, RAS status, tumor sidedness, metastatic sites, and survival outcomes.

## Data Availability

The original contributions presented in this study are included in the article/Supporting Information.
