# Supplementary figures and images for: The ACCEPTance of automation: refining circulating tumor cells enumeration for improved metastatic colorectal cancer prognosis
Source: Mol Oncol. 2025 Sep 19;19(12):3651–64. doi: 10.1002/1878-0261.70126 (PMC12688169; doi:10.1002/1878-0261.70126)

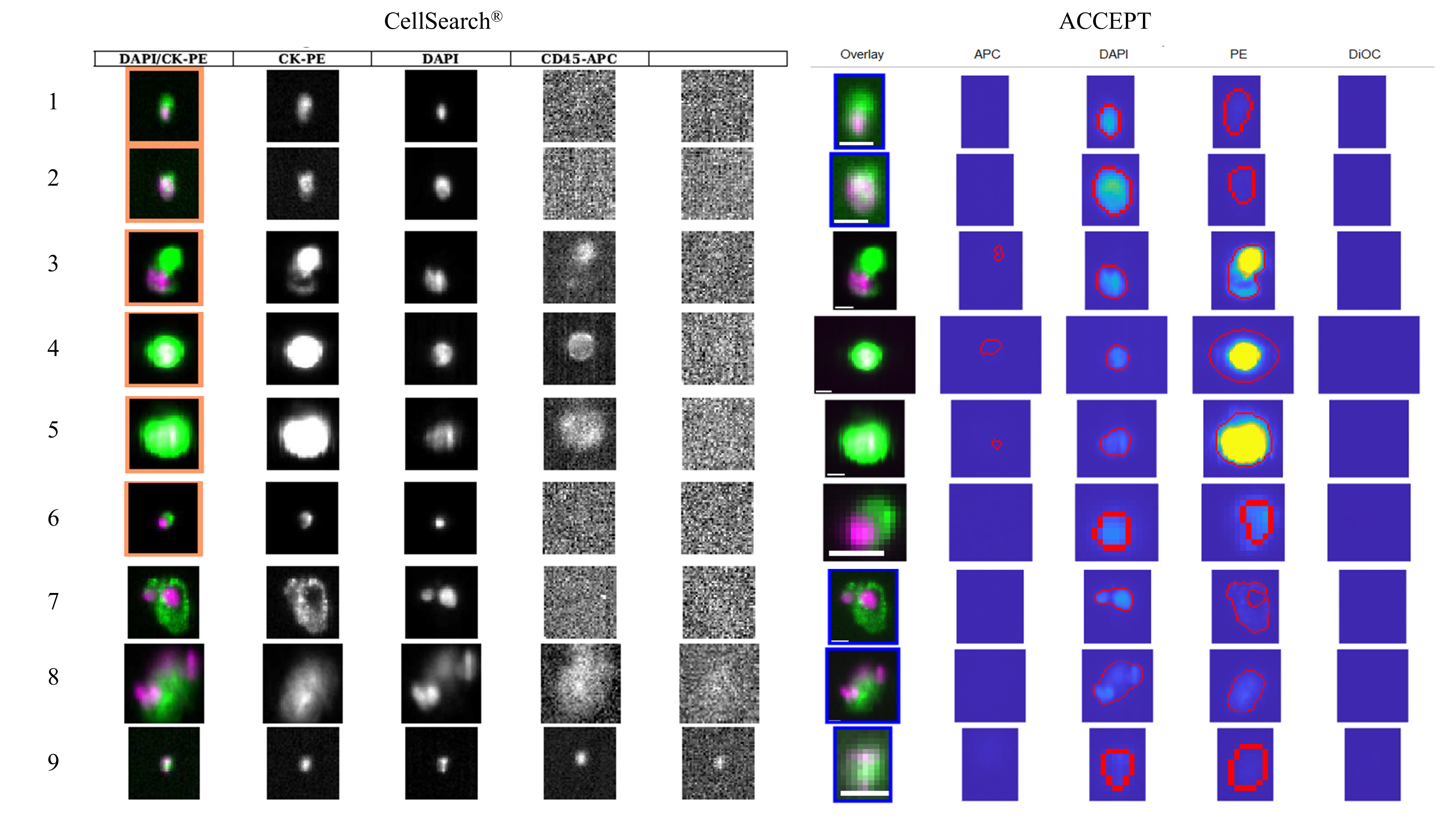

Supplement: Supplementary file 1 — Fig. S1. Representative images demonstrating concordance and discordance in the classification of circulating tumor cells (CTC) by manual (CellSearch®) and automated (ACCEPT) analysis. Panels 1–2: CTC concordantly identified by both CellSearch® and ACCEPT. Panels 3–6: Cells identified as CTC by CellSearch® but not recognized by ACCEPT. Panels 7–9: Cells detected as CTC by ACCEPT but not by CellSearch®. Each image panel includes DAPI (nuclear stain), cytokeratin (CK‐PE), CD45 (APC), and a composite overlay for morphological assessment. APC, allophycocyanin; DAPI, 4′,6‐diamidino‐2‐phenylindole; PE, phycoerythrin. [file MOL2-19-3651-s001.tif]

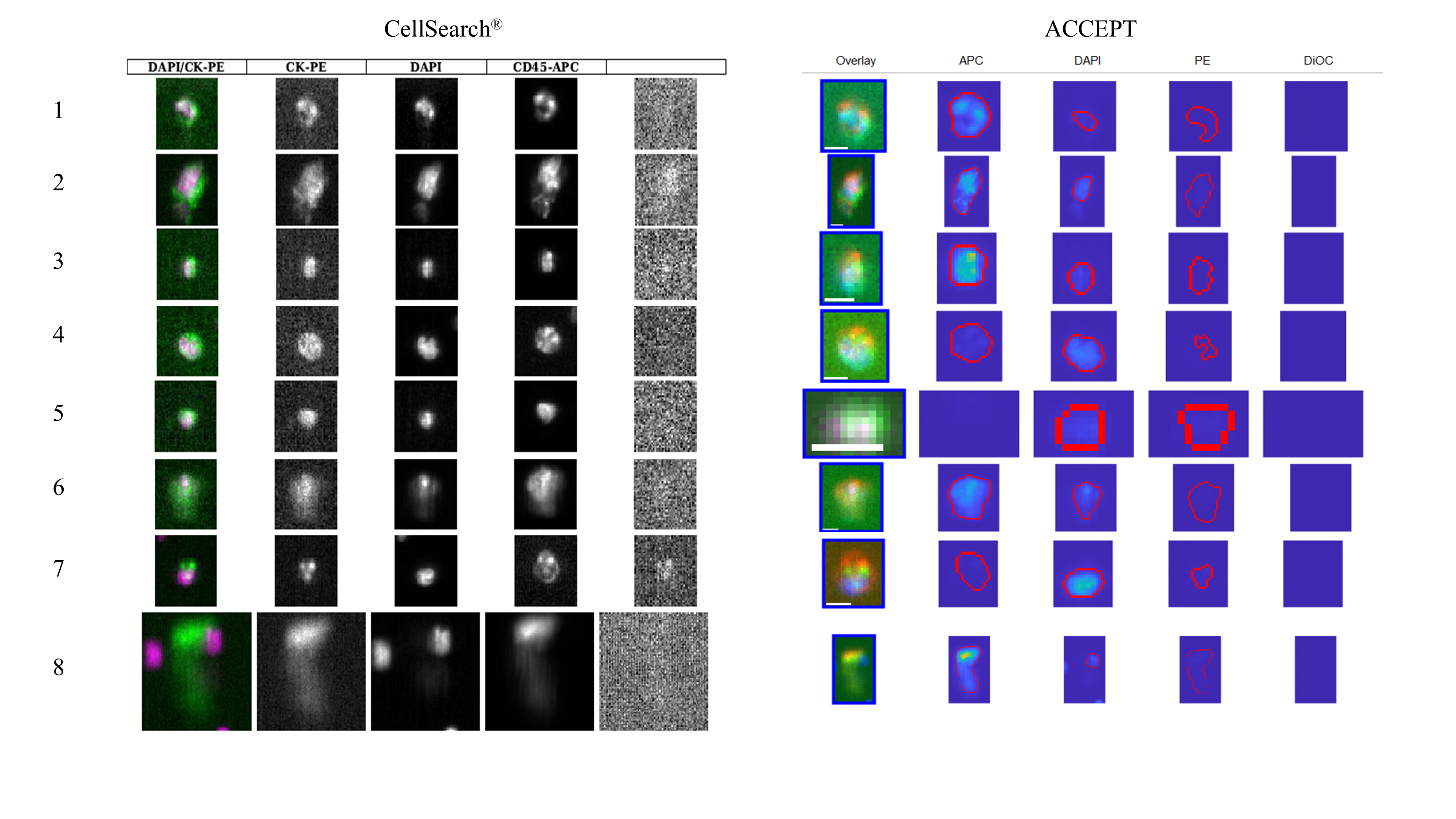

Supplement: Supplementary file 2 — Fig. S2. Representative images demonstrating concordance and discordance in the classification of circulating hybrid cells (CHC) by manual (CellSearch®) and automated (ACCEPT) analysis. Panels 1–4: CHC concordantly identified by both CellSearch® and ACCEPT. Panel 5: CHC identified by CellSearch® but not classified as CHC by ACCEPT. Panels 6–8: CHC detected by ACCEPT but not identified as CHC by CellSearch®. Each image panel includes DAPI (nuclear stain), cytokeratin (CK‐PE), CD45 (APC), and a composite overlay for morphological assessment. APC, allophycocyanin; DAPI, 4′,6‐diamidino‐2‐phenylindole; PE, phycoerythrin. [file MOL2-19-3651-s002.tif]

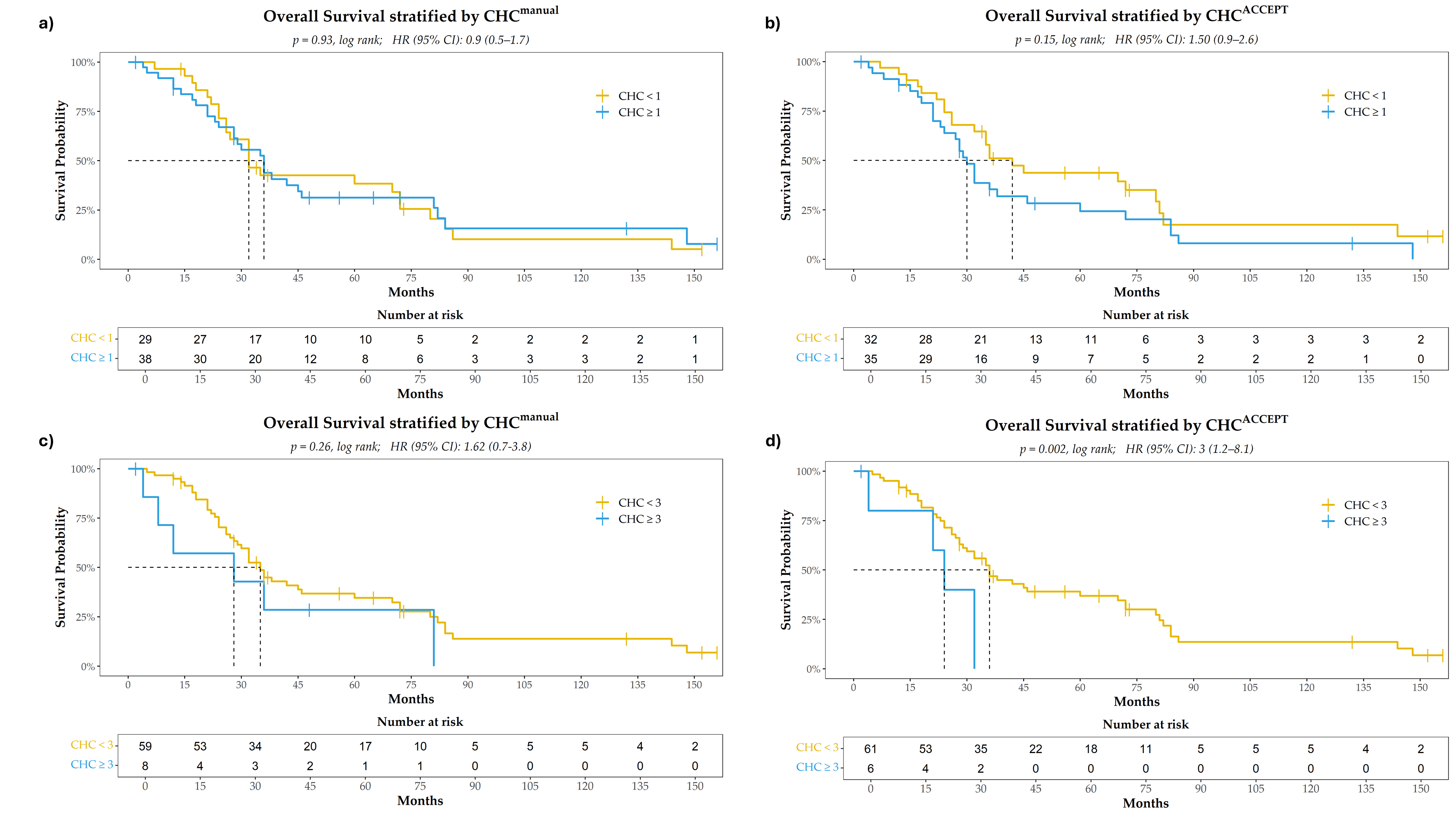

Supplement: Supplementary file 3 — Fig. S3. Kaplan–Meier survival curves for patients (N = 67) stratified by circulating hybrid cell (CHC) count using manual and automated methods. Overall survival stratified by manual (CellSearch®) (a) and automated (ACCEPT) (b) CHC enumeration of patients divided into two groups based on the presence of ≥ 1 CHC. Overall survival stratified by manual (c) and automated (d) CHC enumeration of patients divided into two groups based on the presence of ≥ 3 CHC. For all panels, the log‐rank test was used to assess the differences in survival between groups. Hazard ratios (HR) and 95% confidence intervals (CI) are provided for each comparison. The tick marks indicate censored data. [file MOL2-19-3651-s004.tif]
