# Supplementary material for: The ACCEPTance of automation: refining circulating tumor cells enumeration for improved metastatic colorectal cancer prognosis
Source: Mol Oncol. 2025 Sep 19;19(12):3651–64. doi: 10.1002/1878-0261.70126 (PMC12688169; doi:10.1002/1878-0261.70126)
Supplement: Supplementary file 4 — Table S1. Clinical and molecular characteristics of patients at baseline: CTC and CHC counts, RAS status, tumor sidedness, metastatic sites, and survival outcomes. [file MOL2-19-3651-s003.docx]

**Table S1.** **Clinical and molecular characteristics of patients at baseline:** CTC and CHC counts, RAS status, tumor sidedness, metastatic sites and survival outcomes

| **Patient** | **CTC^manual^** | **CTC^ACCEPT^** | **CHC^manual^** | **CHC^ACCEPT^** | **PFS**  **(mo)** | **OS**  **(mo)** | **Age** | **Sex** | **Tumor sidedness** | **RAS status** | **Site of metastases** |
| --- | --- | --- | --- | --- | --- | --- | --- | --- | --- | --- | --- |
| 1 | 1 | 1 | 1 | 1 | 18 | 29 | 70 | M | Left | wt | peritoneum |
| 2 | 0 | 0 | 1 | 0 | 12 | 82 | 73 | M | Left | wt | liver, lung |
| 3 | 4 | 0 | 1 | 1 | 6 | 18 | 33 | M | Right | mut | bone, lung, peritoneum |
| 4 | 1 | 0 | 0 | 0 | 11 | 72 | 67 | M | Rectum | wt | lung |
| 5 | 0 | 0 | 0 | 1 | 48 | 84 | 64 | F | Rectum | wt | liver, lung, peritoneum |
| 6 | 0 | 0 | 12 | 0 | 13 | 36 | 50 | M | Right | mut | liver, lung |
| 7 | 0 | 0 | 1 | 0 | 35 | 35 | 50 | M | Right | mut | liver |
| 8 | 0 | 1 | 0 | 0 | 46 | 70 | 55 | M | Left | mut | liver |
| 9 | 6 | 4 | 1 | 1 | 10 | 30 | 42 | F | Right | mut | liver |
| 10 | 2 | 1 | 0 | 1 | 21 | 72 | 49 | F | Left | mut | liver, lung |
| 11 | 4 | 3 | 1 | 1 | 2 | 5 | 34 | M | Right | mut | liver |
| 12 | 1 | 1 | 2 | 2 | 4 | 12 | 65 | M | Rectum | mut | lung |
| 13 | 0 | 2 | 0 | 1 | 9 | 17 | 79 | F | Right | wt | peritoneum |
| 14 | 0 | 1 | 1 | 1 | 18 | 84 | 69 | F | Rectum | wt | lung |
| 15 | 0 | 0 | 0 | 0 | 20 | 26 | 58 | M | Right | mut | peritoneum |
| 16 | 2 | 3 | 0 | 1 | 9 | 15 | 64 | F | Left | mut | liver |
| 17 | 0 | 0 | 0 | 0 | 152 | 152 | 51 | M | Left | wt | peritoneum |
| 18 | 3 | 2 | 0 | 1 | 13 | 32 | 72 | M | Left | mut | liver, lung |
| 19 | 0 | 2 | 1 | 1 | 5 | 36 | 81 | F | Left | mut | liver, peritoneum |
| 20 | 0 | 0 | 2 | 0 | 43 | 56 | 65 | M | Right | mut | bone, lung |
| 21 | 0 | 1 | 1 | 0 | 7 | 36 | 72 | M | Left | wt | liver |
| 22 | 0 | 0 | 0 | 1 | 65 | 86 | 57 | F | Left | mut | liver, lung, peritoneum |
| 23 | 0 | 3 | 0 | 0 | 5 | 14 | 67 | M | Left | mut | liver |
| 24 | 0 | 1 | 6 | 0 | 6 | 12 | 60 | M | Right | mut | liver, lung |
| 25 | 3 | 8 | 0 | 0 | 9 | 35 | 75 | M | Left | wt | liver |
| 26 | 0 | 0 | 4 | 0 | 9 | 81 | 60 | F | Left | mut | liver, lung |
| 27 | 0 | 1 | 2 | 1 | 9 | 23 | 43 | M | Rectum | mut | liver |
| 28 | 0 | 0 | 1 | 2 | 18 | 46 | 76 | M | Rectum | mut | lung |
| 29 | 1 | 1 | 3 | 1 | 8 | 48 | 53 | F | Rectum | mut | liver |
| 30 | 17 | 15 | 0 | 0 | 6 | 22 | 72 | F | Right | mut | liver |
| 31 | 0 | 0 | 0 | 0 | 43 | 73 | 50 | M | Rectum | wt | liver |
| 32 | 1 | 1 | 0 | 4 | 11 | 24 | 72 | M | Left | mut | liver |
| 33 | 0 | 0 | 0 | 0 | 23 | 34 | 51 | M | Rectum | mut | lung |
| 34 | 0 | 4 | 1 | 2 | 5 | 28 | 47 | F | Left | mut | liver |
| 35 | 0 | 0 | 0 | 0 | 22 | 37 | 70 | M | Right | mut | liver, lung |
| 36 | 0 | 2 | 2 | 4 | 12 | 21 | 75 | F | Right | wt | liver, lung |
| 37 | 0 | 0 | 1 | 0 | 6 | 24 | 77 | M | Rectum | wt | liver |
| 38 | 1 | 1 | 0 | 0 | 16 | 32 | 66 | F | Right | mut | liver, lung |
| 39 | 2 | 2 | 0 | 0 | 3 | 7 | 64 | M | Rectum | wt | bone |
| 40 | 34 | 36 | 1 | 2 | 4 | 21 | 68 | F | Rectum | mut | lung |
| 41 | 0 | 0 | 0 | 1 | 5 | 27 | 67 | M | Rectum | mut | liver, lung |
| 42 | 1 | 0 | 3 | 2 | 6 | 8 | 61 | M | Right | wt | liver |
| 43 | 1 | 1 | 1 | 0 | 20 | 45 | 74 | M | Right | wt | lung |
| 44 | 1 | 1 | 0 | 0 | 11 | 24 | 70 | M | Left | wt | liver, lung |
| 45 | 1 | 1 | 0 | 3 | 15 | 32 | 69 | M | Left | mut | lung |
| 46 | 0 | 0 | 1 | 1 | 29 | 148 | 60 | M | Right | wt | lung |
| 47 | 0 | 0 | 1 | 2 | 12 | 36 | 60 | M | Left | wt | liver |
| 48 | 1 | 0 | 2 | 0 | 6 | 14 | 65 | M | Left | mut | liver, peritoneum |
| 49 | 0 | 0 | 2 | 0 | 22 | 65 | 73 | M | Left | mut | liver |
| 50 | 0 | 0 | 2 | 1 | 13 | 38 | 67 | M | Rectum | mut | liver, lung |
| 51 | 0 | 2 | 0 | 4 | 17 | 32 | 56 | M | Rectum | mut | liver, lung |
| 52 | 0 | 0 | 3 | 2 | 3 | 28 | 51 | F | Left | mut | liver, lung |
| 53 | 3 | 2 | 0 | 0 | 17 | 80 | 65 | M | Right | wt | liver |
| 54 | 0 | 2 | 1 | 0 | 18 | 156 | 74 | M | Left | wt | lung |
| 55 | 21 | 35 | 2 | 0 | 14 | 72 | 55 | M | Right | wt | liver |
| 56 | 2 | 2 | 0 | 0 | 7 | 26 | 69 | F | Left | mut | lung, peritoneum |
| 57 | 5 | 3 | 2 | 0 | 6 | 17 | 65 | M | Right | mut | liver |
| 58 | 0 | 0 | 2 | 1 | 84 | 132 | 68 | F | Left | wt | liver |
| 59 | 0 | 1 | 1 | 0 | 13 | 42 | 60 | F | Right | wt | liver, lung, peritoneum |
| 60 | 11 | 10 | 1 | 2 | 2 | 12 | 58 | M | Rectum | mut | bone, lung |
| 61 | 1 | 2 | 0 | 1 | 16 | 60 | 53 | M | Right | wt | liver |
| 62 | 0 | 0 | 0 | 1 | 14 | 21 | 64 | M | Rectum | wt | liver |
| 63 | 0 | 0 | 1 | 1 | 25 | 28 | 57 | F | Right | wt | liver |
| 64 | 1 | 0 | 0 | 0 | 4 | 18 | 59 | F | Left | wt | liver, lung, peritoneum |
| 65 | 970 | 842 | 17 | 18 | 2 | 4 | 65 | M | Right | mut | liver |
| 66 | 1 | 0 | 4 | 4 | 4 | 2 | 51 | F | Right | mut | lung |
| 67 | 0 | 0 | 0 | 0 | 144 | 144 | 55 | F | Left | wt | liver |

*CHC: circulating hybrid cells; CTC: circulating tumor cells; mo: months; mut: mutated; OS: overall survival PFS progression-free survuival; wt: wild-type*
